# DNA Markers Unlock Hidden Plant Biodiversity

**DOI:** 10.3390/ijms27135671

**Published:** 2026-06-23

**Authors:** Luigi De Masi

**Affiliations:** National Research Council (CNR), Institute of Biosciences and BioResources (IBBR), Via Università 133, 80055 Portici, Italy; luigi.demasi@ibbr.cnr.it

At present, the molecular genetic characterization of plant bioresources has become essential to improve the conservation and utilization of species and varieties, promote breeding programs, and support origin traceability [1,2,3,4,5]. Molecular markers are discrete DNA sequence regions, revealed through molecular biology techniques, currently used for the analysis of DNA variations and genome architecture. Molecular marker technology (MMT) has greatly enhanced both our capacity to characterize plant genetic variation and to innovate plant breeding. MMT can indeed allow for the identification of specific chromosomal regions associated with genes for traits of interest to transfer to improved varieties via marker-assisted selection (MAS) programs. MMT also has broad potential for detecting plant evolutionary and genetic relationships. Moreover, they can prove useful in the conservation of the biodiversity of natural and domesticated plant populations. Biodiversity plays an essential function in sustainable productions and ecosystem services, strengthening the capacity to adapt to climate change and to biotic stresses, ultimately ensuring food security [6,7]. Moreover, MMT allows the molecular characterization of natural populations and germplasm collections to properly protect them from genetic erosion for future generations and to identify potentially useful genes, new allele combinations, and rare/endangered genotypes. Therefore, DNA polymorphism analysis is of paramount significance for understanding natural variation and conducting breeding in plants.

MMT offers a valuable characterization tool to acquire new knowledge regarding species of agricultural and/or ecological interest, whose strength lies in the fact that MMT is not influenced by environmental factors or plant developmental phases. Today, most research is performed using Next-Generation Sequencing (NGS) technologies to analyze plant diversity at the highest possible genetic resolution. The bioinformatic analysis of genome sequences allows us to understand the effect of DNA variations on gene function and regulation, on the related phenotype, and on evolutionary history in plants. In the current “omics era”, molecular markers as a means of plant diversity characterization have been boosted by the huge quantity of sequence information available. For these reasons, we are now living in a particularly exciting period for molecular genetic studies on plant biodiversity.

In this Special Issue entitled “Plant Biodiversity and Molecular Marker Technology: Discovery and Application of DNA Polymorphisms”, 14 original research articles were collected, providing new perspectives on molecular markers, above all including modern MMT applied to discover and utilize DNA polymorphisms in different plant bioresources of scientific and economic interest. As a demonstration of the broad interest in this topic, the studies come from diverse regions of the world, primarily from Asia (contributions 1–9) to Europe (contributions 10–12), followed by South America (contribution 13) and Oceania (contribution 14). MMT was applied to detect DNA variations in a wide range of plant species, from crops (contributions 3–5, 7, 12–14) to weeds (contribution 9), from aromatic and medicinal (contributions 1, 3, 6, and 8) to fiber plants (contribution 8) and species of forest, forage (contributions 2 and 10), or ornamental interest (contributions 3 and 11). A brief review of the works in this Special Issue follows, with the aim of grouping them by MMT application on specific sectors of interest and highlighting their main outcomes.

Among plant bioresources, the tea plant (*Camellia sinensis* (L.) Kuntze) has considerable commercial and health value due to its high content of antioxidant compounds [8], mainly depending on the tea species/cultivar to which it belongs. Therefore, it would be desirable to obtain molecular markers that can effectively identify tea varieties, perform marker-assisted selection, and map quantitative trait loci (QTLs) for traits of interest. In this regard, in their study, Shen et al. (2024) (contribution 1) developed DNA markers based on Intron Length Polymorphism (ILP) to successfully characterize six *Camellia* species. These ILP markers also showed cross-species transferability (85%) in 11 plant species from six taxonomic families. Finally, a total of 176 plants of *C. tetracocca* were characterized by using 40 ILP markers for assessing genetic diversity and population genetic structure. This research presented an indispensable molecular tool for exploiting genetic diversity within and between the tea species to support genetic conservation and MAS programs, thereby benefiting the tea industry. Furthermore, in the article by Freitas et al. (2025) (contribution 13), elite cocoa clonal cultivars critical to the industry in Brazil were evaluated using their genetic, agronomic, and organoleptic profiles. The goal was to provide a scientific basis for using these cultivars in breeding programs to address global challenges like climate change and new diseases. The molecular findings based on 192 Single-Nucleotide Polymorphism (SNP) markers indicated that they derived mainly from crosses involving Amelonado, Contamana, Iquitos, and Criollo varieties. Significant genetic differences were detected among the cultivars also showing high yield, disease resistance, and optimal characteristics of cocoa beans. This valuable combination is essential for revitalizing cocoa production and planning breeding programs. In another study, Cai et al. (2025) (contribution 6) addressed the challenges of quality control for ginseng (*Panax ginseng* C.A.Mey.) in Traditional Chinese Medicine (TCM) by proposing an evaluation system based on whole-genome, chloroplast genome, and ITS2 DNA barcode variations. The comparative analysis of chloroplast genomes showed SNP mainly located within the large single-copy (LSC) region, as a potential hotspot for ginseng diversity. The conservation of the ITS2 region limited its utilization, while whole-genome analysis revealed more effective inter-simple sequence repeat (ISSR) markers usable to assess the ginseng diversity. By analyzing genetic variations alongside chemical markers, a more reliable way to ensure consistency in ginseng products of TCM was developed. Genetic polymorphism detected by ISSR markers proved to be a powerful predictor of quality, providing a robust new framework for TCM standardization in ginseng.

MMT was also applied to clarify the process of early abscission in macadamia (*Macadamia integrifolia* Maiden & Betche), which refers to the premature separation of fruitlets from the mother plant, negatively affecting crop yield. In this regard, De Silva et al. (2024) (contribution 14) identified the male parent of cultivar “816” embryos by analyzing SNP in their genomic DNA with a customized Single Allele Base Extension Reaction (SABER) system combined with the MassARRAY platform. The macadamia trees retained cross-pollinated fruits rather than those from self-pollination. Cross-pollinated parents positively affected final fruit size and quality compared with the remaining self-fertilized fruits. The selective abscission and the low nut quality of self-fertilized fruits, as determined by DNA analysis, underline the importance of cross-pollination for macadamia productivity. Similarly, Carracedo et al. (2025) (contribution 12) carried out an interesting study on the molecular identification of the West Indian horticultural race and its hybrids of avocado (*Persea americana* Mill.). Avocado fruit is increasingly demanded worldwide for its high nutritional value. It is cultivated from tropical to subtropical regions, and Spain, particularly the Canary Islands, is the leading European producer. In Spain, avocado cultivation requires the genetic identification of West Indian rootstocks since the mother plant determines tolerance to diseases and to climate and soil adversities. Therefore, in this study, a set of Retrotransposon-Based Insertional Polymorphism (RBIP) assays was developed by multiplex PCR and carried out on 58 avocado cultivars. The findings detected the West Indian component in pure cultivars and hybrids of avocado. These molecular tools provide a valuable application of DNA polymorphisms for identification and breeding programs of avocado genetically related to the West Indian race.

Moreover, in the context of forestry biodiversity, the study by Sikora and Celiński (2024) (contribution 10) utilized genome skimming and multilocus DNA barcoding to investigate the evolutionary and taxonomic relationships in the *Pinus mugo* species complex (PMSC). The latter group consists of closely related forest pine taxa, difficult to distinguish taxonomically and widely distributed in European mountain ranges. Highly distinctive genomic regions, up to twenty times more variable, allowed the identification of divergence hotspots that greatly improved the resolution and discrimination power in PMSC. The study also found that *Pinus × rhaetica* does not belong to the PMSC, and it is likely a stable hybrid of *P. sylvestris* and *P. mugo*. In addition, *P. mugo* and *P. uncinata* appear to be involved in the origin of *P. uliginosa* and *P. rotundata*.

Regarding a tuber species of important horticultural and nutritional interest, the study by Zhang et al. (2025) (contribution 4) investigated the genetic mechanisms driving the diverse corolla colors of flowers within the *Solanum* section *Petota* (*Solanaceae*), which includes both wild and cultivated potatoes. By relating recombinant analysis, metabolomics, and transcriptomics, a new genetic locus responsible for corolla coloration was identified in the key regulatory gene StMYB180, shedding light on potato evolution. Pigments showed marked differences between flower variants: purple corollas are characterized by important levels of anthocyanins, whereas yellow corollas show a high accumulation of carotenoids. The study clarifies the mechanisms governing anthocyanin biosynthesis in potatoes and how floral color diversity evolved among wild and domesticated potato relatives. Indeed, the phylogenetic analysis of the corolla color-regulatory genes suggests a strong correlation with the geographic origin and evolutionary history of potato species within the *Petota* section. Another income-producing species, *Brassica napus* L., also known as rapeseed, family *Brassicaceae*, is cultivated for oil-rich seeds and edible roots. Interspecific hybridization between *B. napus* and related genera is explored for rapeseed genetic improvement. *Isatis indigotica* Fort. (Chinese woad) is a highly interesting wild relative used as a genetic resource for dye and medicinal properties. Guo et al. (2025) (contribution 5) successfully identified fragments of alien chromosomes and traced genes for bioactive compounds of *I. indigotica* in intergeneric hybrid offspring with *Brassica napus* L. utilizing the Anchor Mapping of Alien Chromosomal (AMAC) fragment localization method. AMAC will help accelerate the MAS of superior rapeseed varieties by providing a precise map of introgressed regions. A study on rice (*Oryza sativa* L.) focused on P-type pentatricopeptide repeat (PPR) proteins, involved in the processes of the post-transcriptional control of gene expression. Two rice PPR proteins, YS1 and YS2, were identified by Sun et al. (2025) (contribution 7) as essential factors for chloroplast RNA maturation. Using genetic and molecular tools, they showed that mutations in either YS1 or YS2 caused severe defects in rice seedling development. These proteins are required for the correct splicing of the chloroplast *petB* and *petD* introns by directly binding to the transcript of the *psbH-petB* intergenic region. The p*etB* and *petD* genes encode core components of the cytochrome b6f complex, which is essential for electron transport during photosynthesis. Defective intron splicing reduces mature transcript levels, disrupting the assembly and activity of the photosynthetic machinery. Overall, the study concluded that YS1 and YS2 are critical RNA-processing factors that safeguard chloroplast development by ensuring the proper maturation of *petB* and *petD* transcripts. These findings improve our understanding of organellar RNA metabolism to control photosynthetic efficiency and seedling development in rice.

In a study on a tree species of high nutritional, medicinal, ornamental, and ecological importance in China, He et al. (2025) (contribution 3) used phylogenomic analyses to investigate the evolutionary origin of the genus *Xanthoceras* Bunge. Nuclear DNA identified *Xanthoceras* as related to subfam. *Hippocastanoideae*, while plastid DNA placed it as a more anciently diverged lineage of *Sapindaceae*. Transcriptomes from 41 *Sapindaceae* samples were analyzed to investigate this discordance. Analyses provided evidence that *Xanthoceras* underwent ancient introgression, acquiring part of its genetic background from ancestral subfam. *Sapindoideae* lineages. To support this evolutionary origin, morphological analysis also reflected characteristics of both contributing subfamilies. This study showed an example of introgression-driven speciation in angiosperms in which hybridization events played a key role in shaping genetic diversity and evolutionary history of the genus *Xanthoceras*. Moreover, with an innovative approach, Betto et al. (2025) (contribution 11) conducted genotyping and ploidy testing of 96 clonal lines of *Lantana camara* L. by double-digest Restricted site-Associated DNA sequencing (ddRADseq) aimed at variety selection and protection. *L. camara* is a plant of the *Verbenaceae* family, native to the Americas and with high invasiveness potential, known for its ornamental interest as well as for its antimicrobial and antioxidant activities in traditional medicine. In this study, the ddRADseq revealed high homozygosity, but differentiated lantana clonal lines and characterized the germplasm collection for breeding purposes. The molecular profiles also allowed for an estimation of ploidy levels with high accuracy. These findings strongly suggest using high-throughput sequencing techniques for genotyping and breeding programs in precision agriculture.

Natural grass species often show multifunctional interests; this is the case for *Leymus chinensis* (Trin.) Tzvelev, or false wheatgrass. This grass species is a major forage for livestock in Eastern Eurasian grasslands and is currently considered for ecosystem stability, restoration of degraded grasslands, and resistance to drought, salinity, and cold. The study by Ou et al. (2025) (contribution 2) provided valuable molecular tools for the identification, conservation, and breeding of superior varieties in this species. By developing highly informative SSR markers and constructing genetic fingerprints for 105 accessions, the research improved the management of genetic resources and supported the creation of a germplasm database. These molecular findings can boost practical agricultural and industrial applications of *L. chinensis*. An interesting application of MMT was carried out by Li et al. (2025) (contribution 9) on genetic diversity in 46 populations of barnyard grass (*Echinochloa* spp.) infesting paddy fields in Ningxia (China). Native to tropical Asia, barnyard grass is a noxious agricultural weed of the family *Poaceae* and is widespread throughout the world. Its high morphological variability makes it difficult to identify and control. In this study, the barnyard grass diversity was analyzed by DNA barcodes, Start Codon-Targeted (SCoT) markers, and morphological variations. Multilocus DNA barcodes were successfully used to assess the genetic diversity of barnyard grass. *ITS* showed the strongest potential in species identification by single-gene barcoding. Cluster analyses based on *ITS* barcodes and SCoT markers were consistent in grouping 46 populations into five main species groups: *E. colona*, *E. crus-galli* var. *formosensis*, *E. crus-galli*, *E. oryzoides*, and *E. crus-galli* var. *zelayensis*. Morphological traits and genetic profiles of *Echinochloa* populations showed correlation with herbicide sensitivity. The MMT application, consistent with traditional identification systems, highlights the need for an innovative approach to manage this weed.

Traditionally, *Cannabis sativa* L. is classified into three chemotypes based on its profiles in tetrahydrocannabinol (THC) and cannabidiol (CBD): drug-type (high THC and low CBD) used for recreational and medicinal purposes; fiber-type (hemp) (low THC and high CBD) used for textiles and feed; intermediate-type (with high presence of both THC and CBD). For these reasons, accurate classification is legally and commercially essential. However, chemical analysis requires the plant to reach the flowering stage to produce enough cannabinoids for testing. Boonjing et al. (2025) (contribution 8) developed a DNA-based PCR detection method that targets two specific enzymes responsible for cannabinoid production: THCAS (tetrahydrocannabinolic acid synthase) and CBDAS (cannabidiolic acid synthase). By analyzing the genes encoding these two enzymes, this research predicted the chemical profile of future plants before they reached maturity. A phylogenetic analysis based on these two genes classified 85 samples of 46 *Cannabis* cultivars into the corresponding chemotypes. This study successfully examined the relationship between genotype and chemotype in *C. sativa* L., paving the way for molecular detection in *Cannabis* cultivation and production.

Thus, considering all of these investigations, this Special Issue presents advances in plant biodiversity as seen through the lens of MMT, highlighting and updating new applications in plant DNA variations. We focused on the discovery, advantages, challenges, and applications of DNA polymorphisms as molecular markers. A fundamental objective of these studies was to unravel the complexities of plant species identification and explore how MMT can support plant characterization and contribute to a sustainable future. Nevertheless, further research is needed to broaden our comprehension of the agricultural and/or ecological implications of these valuable findings.
